# Injury Patterns and Associated Demographic Characteristics in Children with a Fracture from Equines: A US National Based Study

**DOI:** 10.3390/children11121443

**Published:** 2024-11-27

**Authors:** Randall T. Loder, Alyssa L. Walker, Laurel C. Blakemore

**Affiliations:** 1 Department of Orthopaedic Surgery and Riley Children’s Hospital, Indiana University School of Medicine, 705 Riley Hospital Drive, Suite 1100, Phase 1, Indianapolis, IN 46202, USA; 2 Department of Orthopaedic Surgery, George Washington University School of Medicine, Washington, DC 20052, USA

**Keywords:** equestrian, injury, child, fracture, horse, demographics, NEISS

## Abstract

Background: Fractures often occur due to equestrian activities with injury patterns varying by age. The purpose of this study was to investigate in detail fracture patterns and associated demographics in children due to equine activities. Materials: The US National Electronic Injury Surveillance System was queried for all injuries with the consumer product code 1239 (horseback riding) from 2000 to 2023. Those <16 years old with fractures were extracted. Statistical analyses were performed with SUDAAN 11.0.01™ software to obtain national estimates. Results: There were an estimated 101,677 patients with a fracture. Girls comprised 72.5% and the patient was discharged from the hospital in 81.5% of cases. Fractures involved the upper extremity in 80,973 (80.0%), the pelvis/lower extremity in 11,794 (11.7%), the spine in 3060 (3.0%), the skull/face in 4321 (4.4%), and the rib/sternum in 940 (0.9%). The humerus, elbow, radius/ulna, and wrist accounted for 62.4% of all the fractures. The youngest age group (0- to 5-year-olds) had more boys and was more likely to be admitted to the hospital. The child was injured due to a fall from the horse in 75.7%, bucked/thrown off/kicked off in 17.0%, with the remaining 7.3% from other mechanisms. Conclusions: This extensive description of fractures in children due to equestrian injuries can be used to determine the effectiveness of future prevention strategies, such as protective equipment and educational programs. It also gives pediatric trauma and orthopedic surgeons an overall view of the types of fractures which occur in children due to equestrian activities.

## 1. Introduction

It is well known that activities between humans and horses can lead to injury [1]. The literature regarding equestrian-related injuries to humans is immense. A PubMed search (19 July 2024) [using the search terms horse and injury and humans] identified 1326 manuscripts. The average weight of a standard horse ranges from 410 to 636 kg (900 to 1400 lbs) and for a working/draft horse, up to 818 kg (1800 lbs) [2]. This weight difference alone places humans at distinct risk of injury while handling/working/riding these animals, which are both trusted partners and a prey species that may kick, strike, or bite [1]. These differences are even more magnified for children, who are smaller and often less experienced in working around horses. Injuries may occur while handling horses on the ground, from falls of the rider and/or horse while mounted, or from horse-drawn vehicles. Riders may also be dragged by a frightened horse while an extremity, usually a foot, is caught in the stirrup or other equipment.

Injuries to humans due to equestrian activity can involve major internal organs (e.g., traumatic head injury [concussion, subdural/subarachnoid bleeds, open skull injuries], blunt abdominal [spleen, liver, renal, etc.] and thoracic injuries [pneumothorax, pulmonary and cardiac contusions, etc.]) as well as fractures [3,4,5,6,7], and injury patterns vary by age [5]. The prevalence of fractures in children injured by equine activities ranges from 24% to 37% [3,8,9,10,11], indicating this high burden of fractures in pediatric equine-associated injuries. However, there is no detailed study of fracture patterns specifically in children due to equestrian activities. Knowledge of such patterns would be helpful in guiding future prevention programs/equipment as well as assessing the effectiveness of such endeavors. The purpose of this study is to define the injury patterns and associated demographics of children with fractures due to equestrian activity.

## 2. Materials and Methods

The data for this study were obtained from the National Electronic Injury Surveillance System (NEISS) database. The NEISS is managed by the US Consumer Product Safety Commission (USCPSC) and collects injury data from ~100 hospitals in the United States and its territories having an emergency department (ED). Patient information is collected daily from each NEISS hospital for every patient treated in the ED due to an injury. Estimates from the NEISS database can then be calculated using a statistical methodology to extrapolate from the sampled cases to estimate the total number of injuries for a given population. The database includes the date of visit; sex, age, and race of the patient; incident locale; injury diagnosis; anatomic location of injury; disposition from the ED; and hospital size (strata). There is also a column giving narrative details for each of the injuries. The data set involves only ~100 hospitals; it is weighted and stratified by hospital size which is determined by the number of ED visits for each hospital. These data are publicly available online at https://www.cpsc.gov/cgibin/NEISSQuery/home.aspx, accessed on 13 May 2024. Further details regarding the NEISS data and the acquisition of the NEISS data and guidelines for its use are available at www.cpsc.gov/library/neiss.html, accessed on 5 July 2003. The use of these publicly available de-identified data was determined to be exempt by our local Institutional Review Board.

The NEISS was queried for all injuries with the consumer product code 1239 (horseback riding—activity, apparel, or equipment) over the 24-year period 2000–2023. From this group, those with a diagnosis code of 57 (fracture) were extracted. As the NEISS only gives the most severe diagnosis code, the narrative comments were also searched using the Excel command FIND for the terms fx and fract. This was performed as a person with a more severe injury (e.g., subarachnoid hemorrhage, hepatic laceration) could also have sustained a fracture but would not be identified as such since the other injury was more severe. Each case discovered by this search was reviewed and when confirmed that the injury was a fracture, it was also extracted and added to those found using the NEISS code 57. This constitutes the final data analyzed in this study.

Disposition from the ED was classified as discharged or not discharged; those patients transferred from the initial NEISS hospital to another facility were defined as not discharged. The incident locale was classified as occurring at (1) home, (2) farm/ranch, (3) street/highway, (4) other public property, (5) schools, and (6) places of sport/recreation. Race was classified as White, Black, and Other as per the NEISS. As we specifically wished to study these injuries involving children, a cutoff of <16 years old was used to define the pediatric cohort. These age limits have been previously used as most patients ≥16 years of age demonstrate adult fracture patterns and are transitioning into adult activities (e.g., automobile driving) [12,13]. Further analyses by age were performed by creating three different groups: those 0 to 5 years old (pre-kindergarten), 6 to 10 years old (elementary/primary school), and those 11 to 15 years old (early adolescence).

The anatomic location of the fracture was tabulated. Each location was only counted once even if there was more than one fracture at a particular location (e.g., in a patient with multiple lumbar vertebral fractures, the lumbar site was counted only once). Finally, subgroups were created using the narrative comments for each patient’s ED visit. These groups were (1) the patient was bucked, thrown, or kicked off of the horse; (2) the patient was specifically kicked by the horse; (3) the patient was dragged on the ground by the horse; (4) the patient was mounted on the horse, dismounting, mounting, or simply on the ground; (5) the injury was open or closed; (6) the horse was spooked (frightened by an external stimulus); and (7) the patient was injured while at a likely sanctioned event (rodeo, competition, horse show, barrel racing event, etc.).

Due to the stratified and weighted nature of the NEISS data, statistical analyses must account for such a design [14]. SUDAAN 11.0.01™ software (RTI International, Research Triangle Park, NC, USA, 2013) was therefore used. The estimated number of injuries/ED visits is calculated, along with 95% confidence intervals [CIs] of the estimate. These methods are well described [14,15]. When the actual number of patients (n) is <20, the estimated number (N) becomes unstable and should be interpreted with caution; thus, we report both the n and N [15,16]. Continuous data are expressed as the mean and categorical data as frequencies and percentages. Differences between continuous variables were determined using the t-test or ANOVA. Differences between categorical variables were determined with the χ^2^ test. Differences over time (years) were analyzed with join point regression analysis (Joinpoint Regression Program, Version 4.8.0.1, April 2020; Statistical Research and Applications Branch, National Cancer Institute [https://surveillance.cancer.gov/joinpoint/, accessed on 4 April 2020]).

## 3. Results

Over the 24-year period 2000–2023 inclusive, there were 34,091 ED visits for equine-associated injuries, or a national US estimate of 1,462,193 patients [1,374,461–1,549,925] as described in the methods. There were an estimated 334,306 [302,674–368,180] < 16 years of age. Of these 1,462,193 patients, there were 101,677 [100,603–102,4125] < 16 years of age with a fracture. Using US Census Bureau data for the years 2000, 2010, and 2020, the average number <16 years of age living in the US for those three years was 62,280,371. These 101,677 fracture patients over the 24 years in the study equate to 4237 children per year, or an incidence of 6.51 per 100,000 children per year.

### 3.1. Overall Demographics

**Figure 1:**
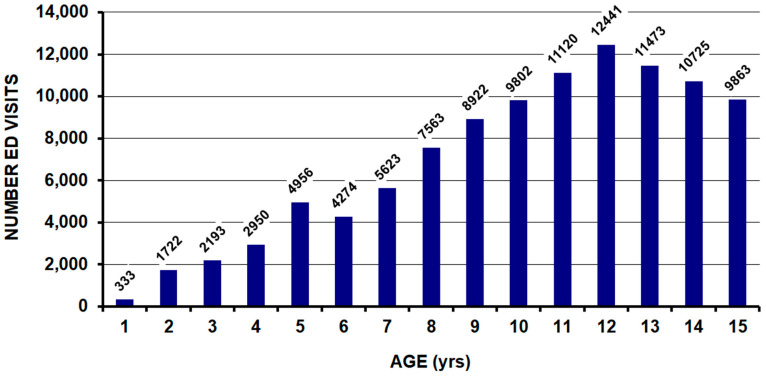
Histogram of all patients. The greatest number occurred in the 12-year-old group.

The average age was 10.3 [10.1–10.5] years; the highest number occurred in 12-year-olds (Figure 1). Girls comprised 72.5% and boys 27.5% of the group (Table 1). Most of the patients were white (92.1%). The patient was discharged from the hospital in 81.5% of cases and admitted to the hospital in 18.5% of cases. There were no fatalities in the ED or DOA cases. The injury occurred at home in 41.9%, at places of sports/recreation in 35.1%, on a farm/ranch in 15.1%, with the remaining 7.9% occurring at other locales. The child was injured due to a fall from the horse in 75.7%, bucked/thrown off/kicked off in 17.0%, with the remaining 7.3% from other mechanisms; 96.6% of these other mechanisms occurred while mounting the horse. In 2.3% of cases, the horse had been spooked, in 2.9%, tack/equipment was directly involved, and in 0.4%, the child was dragged by the horse. An associated internal organ injury occurred in 2.7% of the patients.

### 3.2. Fractures

**Figure 2:**
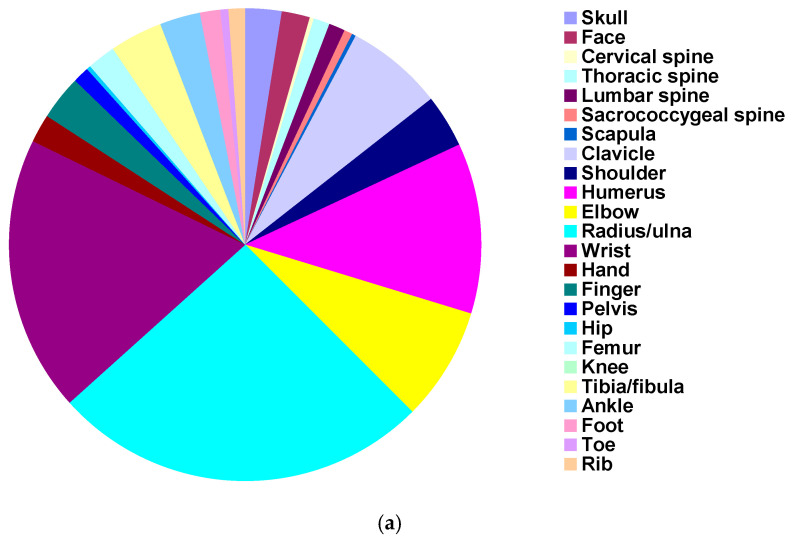

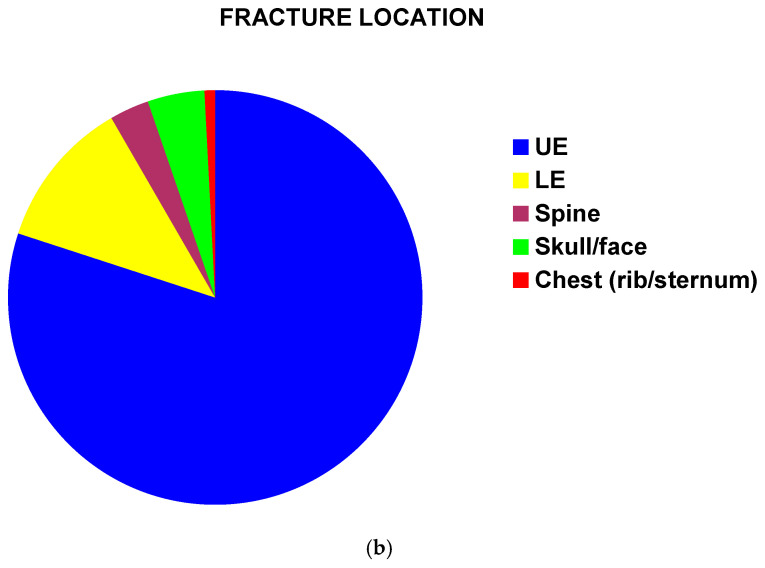
Fracture locations. (**a**) Detailed locations. (**b**) Location aggregated by upper extremity, lower extremity, spine, head/neck, and rib/sternum.

The anatomic location of the fracture was known in 101,174 of the patients (Table 2, Figure 2a,b). The upper extremity was involved in 80,973 (80.0%), the pelvis/lower extremity in 11,794 (11.7%), the spine in 3060 (3.0%), the skull/face in 4321 (4.4%), and rib/sternum in 940 (0.9%). The humerus, elbow, radius/ulna, and wrist accounted for 62.4% of all the fractures. The average age in years for these groups was 10.0 for the upper extremity, 11.7 for the lower extremity, 12.9 for the spine, 9.9 for the skull/face, and 11.3 for the rib/sternum group (*p* < 10^−4^) (Supplemental Table S1). The fracture was open in 1142 [668–1933] (1.1%) of the cases.

**Figure 3:**
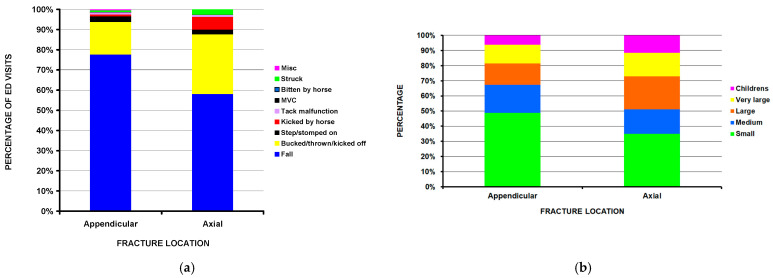
Appendicular compared to axial fracture locations in children with injuries due to equines. (**a**) By mechanism of injury (*p* = 0.021). (**b**) By hospital size (*p* = 0.021).

The fracture locations were aggregated into appendicular and axial groups for further analysis (Table 3), as the number of fractures in many of the detailed locations was small. Axial was defined as the head/neck and spine; the appendicular group comprised the pelvis and all other locations. There were 92,767 appendicular fractures with an average age of 10.2 years and 8437 patients with axial fractures with an average age of 11.2 years; this age difference was significant (*p* = 0.0004). Those patients with axial fractures were more frequently admitted to the hospital (52.7%) compared to the appendicular group (14.4%) (*p* = 0.0003). While the predominant mechanism of injury in both groups was falling off the horse, falling off the horse occurred in 77.7% of the appendicular fracture group and 58.1% of the axial fracture group, while being bucked/thrown/kicked off the horse occurred in 29.6% of the axial fracture group and 16.1% of the axial fracture group (*p* = 0.021) (Figure 3a). The percentage of appendicular fractures seen at small hospitals was 48.7% compared to 34.6% for axial fractures, while axial fractures were seen at children’s hospitals nearly twice as often as appendicular fractures (11.3% vs. 6.1%) (*p* = 0.021) (Figure 3b). 

**Figure 4:**
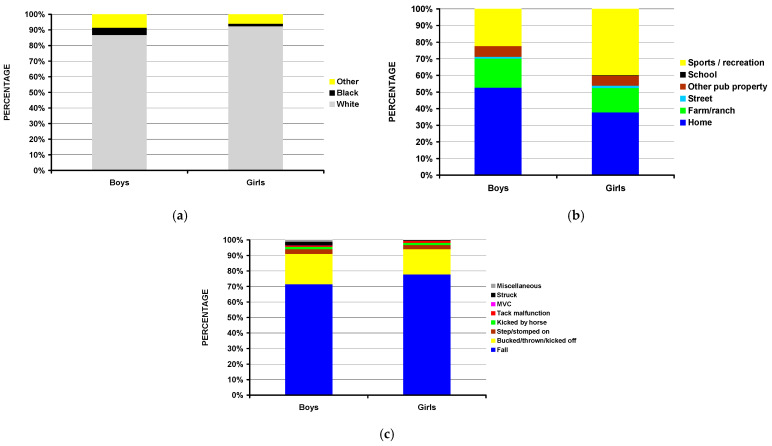
Differences between boys and girls with fractures due to equine activities. (**a**) By race (*p* = 0.003). (**b**) By incident locale (*p* = 0.003). (**c**) By mechanism of injury (*p* = 0.035).

When analyzing differences between boys and girls sustaining fractures due to equestrian activities, several findings were noted (Table 4). Boys were slightly younger than girls (average age 9.9 vs. 10.4 years (*p* = 0.004)). While the majority of the children were White, a higher percentage of boys were Black and from other races, with the percentage of White children being 86.7% vs. 94.0% for boys and girls, respectively (*p* = 0.003) (Figure 4a). The incident location also differed, with boys having more injuries occurring at home compared to girls (52.6% vs. 37.8%) and with girls having more injuries at places of sports and recreation (22.3% vs. 39.8%) (*p* = 0.003) (Figure 4b). While the mechanism of injury for both boys and girls was predominantly due to a fall from the horse, boys had more cases of being bucked/thrown/kicked off the horse compared to girls (19.6% vs. 16.2%) and being stepped on by the horse (3.1% vs. 2.6%) (*p* = 0.035) (Figure 4c).

We analyzed in detail the mechanism of injury between axial and appendicular fractures (Table 2). Appendicular fractures had a higher prevalence of a fall from the horse compared to axial fractures (77.7% vs. 58.1%), while axial fractures had a higher prevalence of being bucked/thrown/kicked off the horse (29.6% vs. 16.1%) (*p* = 0.021). When breaking down by the five major fracture locations (upper extremity, lower extremity, spine, skull/face, and rib/sternum) (Table 5), there were significant differences (*p* < 10^−4^). Fractures of the spine had the highest prevalence of being bucked/thrown/kicked off the horse at 43.4%, followed by the rib/sternum at 33.3%. Upper extremity fractures had the highest prevalence of a fall from the horse (80.5%), followed by the skull/face at 64.5%.

Illustrative case examples from the narrative comments are as follows:

1. A 13-year-old female was riding her horse; the horse tripped and fell backwards on the patient. She was wearing a helmet, and riding a Western saddle, but the saddle horn impaled the vagina, resulting in a vaginal laceration and pelvic fracture.
2. A 13-year-old female was horseback riding at a rodeo event when she flipped off, landing on her head and sustaining a depressed skull fracture along with a subarachnoid hemorrhage.
3. A 13-year-old male was riding a pony when the pony fell landing on the child’s leg resulting in a femur fracture.
4. A 7-year-old girl fell off a pony sustaining a displaced basilar femoral neck fracture.
5. A 9-year-old boy was thrown from a horse and dragged 50 yards, sustaining an open pelvic fracture with a perineal laceration and a right femur fracture.
6. A 13-year-old female was riding a horse when she fell off and then the horse fell on top of her. She sustained a liver laceration, colon contusion, and a closed fracture of the right pubic ramus.
7. A 9-year-old female was thrown off a horse and dragged 30 feet; the leg got tangled in the reins resulting in a tibia fracture, concussion, and scalp hematoma.
8. A 6-year-old female fell off a horse at her grandmother’s farm sustaining an open, comminuted, markedly displaced supracondylar fracture of the humerus.
9. A 5-year-old female fell off a horse sustaining both a fracture of the humerus and clavicle.
10. An 11-year-old female fell off a horse during a riding performance and sustained a right elbow and orbital fracture.
11. A 5-year-old female was riding a horse and got kicked on the leg by another horse resulting in an open tibia fracture.

The narrative comments for the pelvic fractures (48 actual patients, 1131 estimated) were further reviewed. The details of what type of pelvic fracture are not given; however, at least one of the patients sustained an acetabular fracture, quoting, “an 11-year-old female fell off her horse when it began to buck, sustaining an acetabular fracture along with a hip dislocation”. The average age was 12.3 years (range 3–15 years), with 30 being teenagers. There were 40 girls and 8 boys.

### 3.3. Associated Internal Organ Injuries

**Figure 5:**
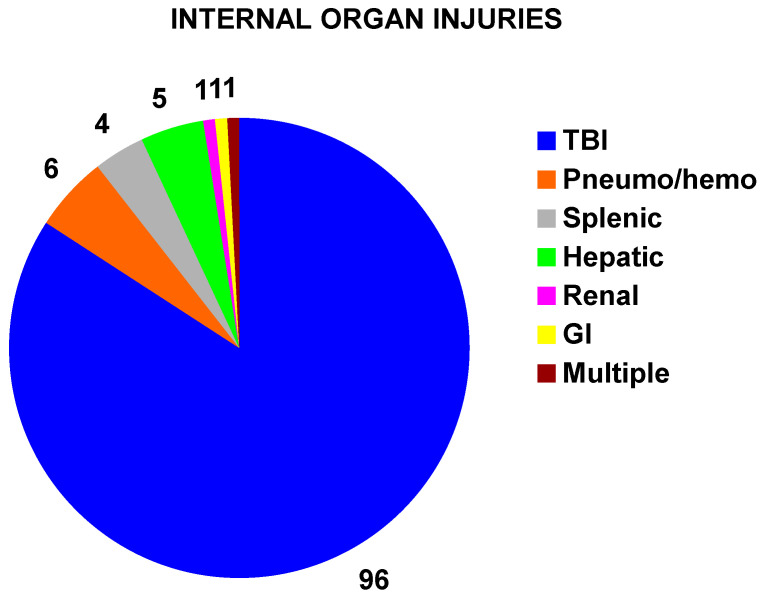
Distribution of associated internal organ injuries in children with fractures. The numbers of each pie slice are the number of patients having each particular internal organ injury. TBI = traumatic brain injury, pneumo/hemo = pneumothoroax/hemothorax.

Only the actual numbers are reported due to the small numbers. There were 114 children with an associated internal organ injury (Figure 5); 96 (84%) had traumatic brain injuries (concussions, other brain injuries [e.g., hemorrhage]). The injury in the remaining 18 patients was a pneumo/hemothorax in 6, splenic injury in 4, hepatic injury in 5, renal injury, gastrointestinal injury, and multiple injuries in 1 patient each. All associated internal organ injuries were in patients who had sustained fractures. There was no difference by sex, with an internal organ injury rate of 3.4% (31 of 911) in boys and 3.6% (83 of 2308) in girls (*p* = 0.79). Those with internal organ injuries were more commonly admitted to the hospital (65 of 114 [57%]) compared to those without internal organ injuries (738 of 3103 [21.6%]) (*p* < 10^−6^) (ED disposition not known in 2 patients). Two illustrative cases of associated internal organ injuries are given.

1. A 15-year-old female was at a hunter-jumper competition. Her horse tripped, and she fell to the ground with the horse falling on top of her. She sustained a crush injury to the chest with a left bronchial transection, a splenic laceration, a proximal humerus fracture, and vertebral body fractures at T8/T9.
2. A 15-year-old female was riding a horse and was bucked off. She sustained a concussion, a lunate fracture/dislocation of the right wrist, and a closed fracture of the left distal radius/ulna.

### 3.4. By Age Groups

**Figure 6:**
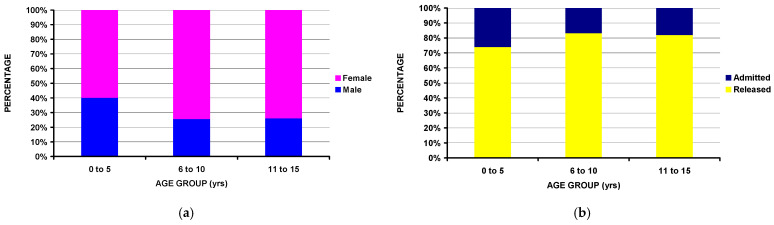

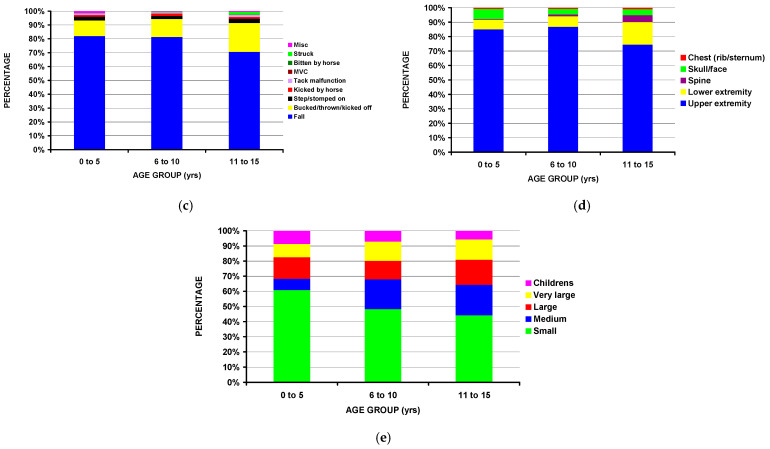
Differences by age groups. (**a**) Differences by sex; note there were more boys among those < 6 years old (*p* < 10^−4^). (**b**) By disposition from the ED (*p* = 0.029). Those <6 years old were more commonly admitted to the hospital. (**c**) By mechanism of injury (*p* = 0.031). Those in the 11 to 15-year-old group had the highest percentage of being bucked/thrown/kicked off the horse. (**d**) By major fracture location (*p* < 10^−4^). Lower extremity fractures were more than twice as frequent in the oldest age group compared to the youngest (15.1% vs. 6.5%). (**e**) By hospital size (*p* = 0.0003).

There were significant differences between the three age groups (Table 6). The youngest age group (0- to 5-year-olds) had more boys (*p* < 10^−4^) (Figure 6a) and was more likely to be admitted to the hospital (*p* = 0.029) (Figure 6). The oldest age group (11- to 15-year-olds) had the greatest prevalence of being bucked/thrown/kicked off the horse (*p* = 0.031) (Figure 6c) and the highest prevalence of lower extremity fractures (*p* < 10^−4^) (Figure 6d). Interestingly, those seen in the smallest hospitals were in the youngest age group (*p* = 0.0003) (Figure 6e).

### 3.5. Temporal Variations

**Figure 7:**
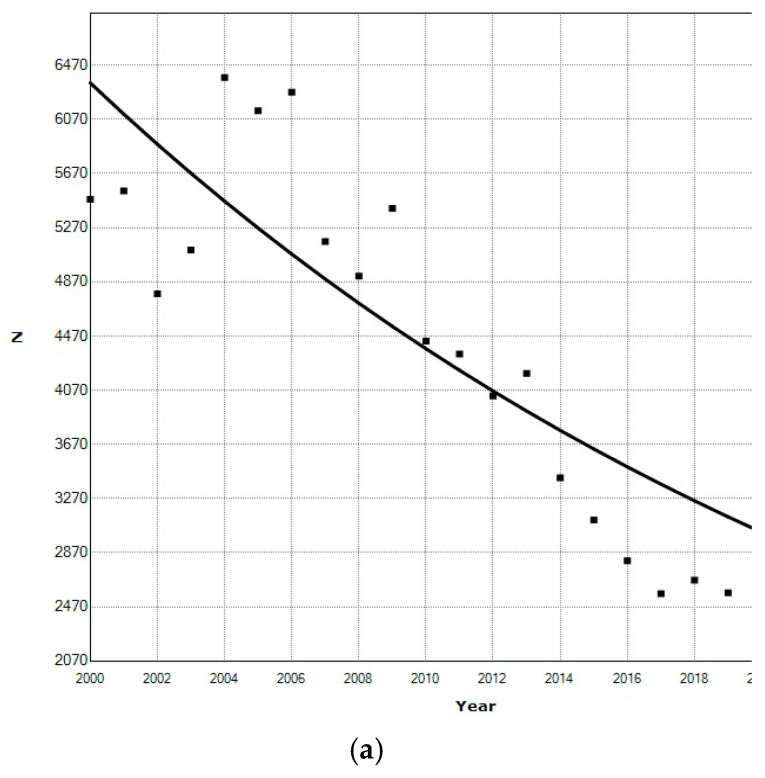

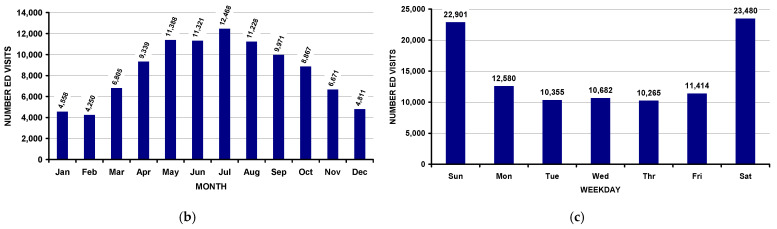
Changes by year, month, and weekday in pediatric equestrian-related fractures. (**a**) Join point analysis demonstrating the average annual percentage decrease of 3.64% in children with fractures due to equine activities from 2000 through 2022 (*p* < 10^−6^). The black squares are the estimated number per year and the line is the best fit joinpoint regression. (**b**) By month of year. The estimated number of ED visits per month is at the top of the bar. (**c**) By weekday. The estimated number of ED visits per weekday is at the top of the bar.

There was an overall annual percentage decrease of 3.64% (*p* < 10^−6^) in ED visits for children with fractures due to equine activities (Figure 7a). More injuries occurred in the summer months (Figure 7b) and on weekends (Figure 7c).

## 4. Discussion

In the present study, fractures accounted for 30.4% (101,677 of 334,306) of all equine-associated injuries in those <16 years of age. This 30.4% is similar to many studies (range 24% to 37%) [3,8,9,10,11], but nearly double that of 16% from Bernhang et al. [17] and less than that of 57.1% from Kiss et al. [18]. The 1992 study of Bixby-Hammett [8] used the NEISS database for those <25 years of age for the years 1979 through 1982 and 1987 through 1990. While an older study compared to ours, the ages are somewhat similar, and our 31.1% is very close to their 25.6% prevalence of fractures. The study of Kiss et al. [18] was a questionnaire study of children seen at their center who had been previously injured in equestrian activities. We surmise that those with more serious injuries were more likely to fill out and return the questionnaire, which could account for their higher percentage of fractures.

The prevalence of fractures due to equestrian injury has been noted to vary by age. In a study of shows sponsored by the American Horse Show Association and the United States Pony Club [17], 15.5% of the injuries in the American Horse Show Association cohort and 32% in the United States Pony Club cohort were fractures. They also saw a higher prevalence of fractures in children less than 16 years of age. Bilaniuk et al. [5] found that fractures accounted for 27% in those 0–18 years old, 26.8% in those 19–49 years old, and 41.9% in those 50 years old or older.

The breakdown of fracture locations in children is limited. Theodore et al. [10] reviewed 171 children with horse-related trauma presenting to a pediatric trauma center in Queensland, Australia, and found that upper extremity fractures accounted for 20.9% of all the injuries. Bilaniuk et al. [5] noted that in the 0–18-year-old group, there were 39 fractures in 145 patients. These involved the upper extremity in 24 (62%), the spine in 7 (18%), the skull/face in 3 (8%), the rib and pelvis in 2 each (5%), and the lower extremity in 1 (2%). Our upper extremity involvement is higher than that of Theodore et al. [10] and Bialinuik et al. [5]; we attribute this to our study covering all EDs and not just those seen at trauma centers. Kiss et al. [18], in a study of 240 pediatric equine injuries, encountered fractures in 64, with 1/2 of the fractures (n = 32) involving the upper extremities.

There are no studies giving detailed fracture locations due to equine injuries in children. We found that 32.1% of all the fractures occurred in the radius/ulna or wrist. Interestingly, our study did show a trend of increasing lower extremity fractures in older children, with lower extremity fractures accounting for 15.1% of all fractures in those 11–15 years of age, but only 6.5% of those 0–5 years of age.

Bernhang et al. [17] noted that 7.7% of all the fractures were open, lower than the 1.1% in this study. We suspect that if a fracture occurred at a horse show, as studied by Bernhang et al. [17], the injury itself might be more severe, as many of the injuries in that study occurred during jumping activities, and such falls would be higher energy than a simple fall off of a horse at a slow gait. Regarding pelvic fractures, Moshieff et al. [19] encountered a 12.9% open fracture prevalence.

Lower limb injuries occurred in 20% of the patients. Altgärde et al. [20], in a Scandinavian study of horse-related injuries, reported 24% lower limb injuries in adults and 19% in children. Lower limb injuries are more frequently due to a kick from a horse or being trodden on the foot [6]. This is supported by Lang et al. [21], who found a significantly greater number of lower extremity injuries in adults compared to children, likely due to the fact that adults are more involved in grooming and feeding. Since our study involved children and the vast majority fell off the horse, our 20% of lower limb injuries likely reflects that few were kicked or trodden upon by the horse.

While extremity fractures can be limb-threatening due to vascular disruption, we found no such cases in the narrative comments. However, the prevalence of open fractures was 1.1%, and while not high, open fractures can have long-term consequences if chronic osteomyelitis develops. This is especially pertinent as equestrian-related open fractures likely occur in highly contaminated environments, such as farms and arenas where dirt and equine feces are present. It is well known that open fractures occurring on farms are at increased risk of infection [22,23], and any open fracture occurring in a farm/agricultural setting is automatically categorized as the most severe (Gustilo-Anderson type III) [24]. Finally, as this is an ED-focused database, long-term outcomes regarding physeal injury due to the fracture are unknown, and physeal injury can lead to significant limb length and/or angular deformity in growing children.

Spine fractures are potentially devastating due to the risk of neural injury. In this study, there were an estimated 3060 spine fractures in 101,174 patients, or 3.0%. Kiss et al. [18], in a study of 240 pediatric equine injuries, noted 8 ligamentous injuries involving the spine, with no mention of neural deficit. In one study of 284 injuries [5] from a trauma center, 79 involved the spine; however, the exact type of injury was not described (fracture, dislocation, etc.). In a study of 315 children with equestrian-related injuries [25], there were eight spine fractures. In addition, there was a child with a C4 dislocation and one child with a spinal cord injury with an eventual return of function. Hessler et al. [26] studied 31 children <18 years of age who had been thrown off a horse and sustained torso injuries. They described seven with thoracic spine fractures and three with lumbar spine fractures.

There are several studies specifically addressing spine injuries from equestrian activity [4,27,28,29,30]. De Jesus et al. [27] described 19 patients with spinal injuries in a study of a neurosurgery database from Puerto Rico. Interestingly, 87% of the patients were male. They noted that horseback riding in the streets of Puerto Rico is much more common among men, riding in a mounted procession called a “cabalgata”. In a study from the UK [28] of 122 patients with 230 spine fractures occurring during sporting activities, the sport most commonly associated with spinal fractures was horse riding (55 of 122–45%), and 60 (49%) were male. These 55 patients sustained 92 spine fractures. In a study from the Netherlands on spine fractures due to horse riding [4], 36 fractures occurred in 32 patients, with the majority being female (28 or 88%) and 2 occurring in children. In an Australian study, Roe et al. [29] described 30 vertebral column fractures due to horse riding in 16 females and 14 males, as well as another 34 patients (21 males, 13 females) with spinal cord injuries. They noted that occupational riders were more often male (86%) while leisure riders were more commonly female (58%). In our present study of an estimated 3060 spine fractures due to equestrian activities in children, the majority (76.0%) were girls (Supplemental Table S1).

This study confirms the high percentage of females participating in equine activities, which is 72.5% in this study and ranges from 70% to 93.7% in other studies [5,6,17,18,31,32,33]. The meta-analysis of Neville et al. [1] noted a female percentage ranging from 52% to 93.7%. We noted that the youngest children in this study (5 years old and younger) had the highest percentage of males (40%), with those 6 years and older being 26% male. This is similar to the findings by Jones [34] in a New Zealand study where there were actually more boys than girls in the 0- to 4-year-old group, and with females in the majority after age 4 years. This same pattern was also seen in an earlier study using NEISS data [3]. This high female percentage reflects the overall high percentage of females in equestrian activities [35,36,37] and represents 79% of the equine industry in Canada [36].

### 4.1. Straddle Injuries, Pelvic Fractures, and Associated Urogenital Injuries

Much of the literature discussing injuries of the bony pelvis due to equestrian activities involves adults, especially males [38,39,40,41]. Pelvic fractures in children, excluding the common athletically related avulsion apophyseal fractures (superior and inferior iliac spine, ischial tuberosity, lesser trochanter) [42,43,44], are rare and are due to high-energy mechanisms [45,46,47,48,49,50]. Gänsslen et al. [45,51] extracted pediatric pelvic fractures from 13 individual studies with an average age of 9 years. The male-to-female ratio was 1.4:1, or 58% male. When using their tabular raw data, motor vehicle crashes accounted for 45.4%, a pedestrian-involved injury in 31.5%, a motor vehicle passenger occupant in 9.7%, a bicycle in 2.6%, a fall in 5.0%, sports-related in 1.1%, farm-related in 0.3%, crushing injuries in 1.2%, and all other mechanisms in 2.6%. The overall mortality rate was 6.4%. Acetabular fractures occurred in 10.4%.

We then also reviewed multiple pediatric pelvic fracture studies looking for specific mention of equestrian injury mechanisms [49,50,52,53,54,55,56,57,58,59,60,61,62,63,64,65,66,67,68], and only 2 of 19 noted that the injury involved a horse [60,65]: 18 patients in the study of Hailer et al. [60] and 3 in the study of Rieger et al. [65]. In the study of Rieger et al. [65] from Germany, 3 of 54 (6%) children were crushed by the horse. In the study of Hailer et al. [60], 18 of 223 children (8.1%) sustained pelvic fractures due to equestrian injury, but the exact details were not given (i.e., bucked off, crushed, etc.). Hailer et al. [60] noted that 17 of the 18 (94%) pelvic fractures from equestrian activity occurred in girls.

In this study, there were 1311 children with pelvic fractures or 1.1% of all equestrian-associated fractures. Many of these were severe, as seen in the illustrative cases: a 13-year-old female sustained both a vaginal laceration and pelvic fracture; a 9-year-old with an open pelvic fracture, perineal laceration, and a right femur fracture; and a 13-year-old female who sustained a liver laceration, colon contusion, and a closed fracture of the right pubic ramus. Of the actual 48 patients, 83% were girls, greater than the 73% overall female percentage. There was one (2%) described acetabular fracture; there may have been more but were not mentioned in the narrative comments. This 2% is less than the 10.4% encountered in the review by Gänsslen et al. [45,51]. We suspect that this is due to the fact that many of the studies reviewed by Gänsslen et al. [45,51] were from trauma centers, which would be biased towards more severe injuries, such as acetabular fractures. These complex injuries are generally the result of a fall of horse and rider, in which the rider remains in the saddle and is pinned beneath the animal. Soft tissue straddle injuries in children are not uncommon and typically are due to a fall from a bicycle or other playground equipment. However, it is not known if all were true straddle injuries or if there were instances in which a fall of the rider alone resulted in a pelvic fracture.

Torode and Zieg [68], in a study of 141 children with pelvic fractures, encountered 8 with perineal/vaginal lacerations, 7 with urethral injuries, and 4 with bladder ruptures. Rieger et al. [65] found in 54 children with pelvic fractures urethral/bladder injuries in 8 (15%), rectal tears in 5 (9%), and vaginal tears in 5 (9%). Moshieff et al. [19], in a study of 15 children (8 boys, 7 girls) who sustained open crush injuries to the pelvis, encountered 10 anorectal tears, 7 urethral/bladder injuries, and 5 vaginal lacerations. Spiguel et al. [67], in 13 children with pelvic fractures, encountered 2 patients (15%) with genitourinary injuries; one girl required a nephrectomy along with repair of perineal/vaginal tears and urethral laceration, and a boy sustained scrotal/testicular trauma requiring an orchiectomy. Delaney et al. [69], in a review of 2639 females from the National Trauma Data Bank Registry under the age of 16 with pelvic fractures, noted a bladder/ureteral injury in 81. Those with a vaginal laceration, disruption of the pelvic circle, multiple pelvic fractures, or a sacral spine injury were at the highest risk for a bladder/urethral injury; eight open fractures were identified. Again, the mechanism was motor vehicle involvement in 73.7% and a fall in 7.7%. None of these studies mentioned equestrian-activity-associated injuries.

In a literature review of pediatric pelvic fractures, Nguyen et al. [47] noted that in boys with a urethral injury due to a pelvic fracture, up to one-half will develop erectile dysfunction in puberty [70]. They also noted that girls with vaginal injuries felt to be minor at the time of fracture can present with dyspareunia and vaginal stricture years later. Fowler et al. [71] described a 26-year-old female with sexual dysfunction and vaginal stricture after a pelvic fracture sustained at the age of 10 years. Thus, as stated by Nguyen [47], pediatric patients should be followed up in the long term to ensure that potential delayed complications can be addressed. The pediatric orthopedist is not usually aware of the long-term issues that may arise from a child sustaining a pelvic fracture along with a urogenital soft tissue/internal organ injury. With this information, pediatric orthopedists should caution parents regarding these potential future issues if there are also associated urogenital/rectal injuries, of course, in concert with the specialist taking care of those non-osseous injuries.

### 4.2. Immediate Outcomes

If hospital admission is used as a proxy for severe injuries, then most of the injuries in this study were not severe. However, this study is only on fractures. We suspect that the admission rate might be greater for injuries to the head and torso. Mutore et al. [72], using data from the National Trauma Data Bank, noted that the risk of hospital admission from equestrian injuries is higher than in football, motor vehicle racing, and skiing. They also stated that equestrian injuries presenting to the ED had a low likelihood (5.15%) of being discharged home; however, this is a skewed database as it only involves designated trauma centers. Such centers in this study would only be found in large and very large hospital sizes. In this study, 65.1% of the patients were seen in small- and medium-sized hospitals. While there were some associated internal organ injuries, isolated injuries to the head/chest/abdomen without a fracture were excluded, and those likely would have a higher admission rate even in the NEISS data.

In a recent literature review of horse-related injuries [1], the mortality rates were between 0.6 and 1.0% for all studies. The most common critical injuries were traumatic brain injuries (74.83%), thoracic injuries (18.44%), abdominal injuries (5.31%), and 2.19% extremity injuries. Pounder et al. [73], in Australia, studied 15 deaths due to trauma in horse riders and calculated that the rate was 1 per million population per year. This is supported by other studies [33,74].

In our study, there were no fatalities in the ED or DOA patients. However, as this is an ED database, there may have been fatalities that occurred later during a hospital admission. Also, the NEISS stopped recording fatalities in the ED in 2020. There was one recorded horse fatality, as described in the illustrative spine fracture section above. The mortality rate of pediatric equestrian injuries is low in the literature. We were able to extract information from six studies specifically addressing children [11,20,25,32,75]; there were 12 deaths in these 1438 children, or 0.8%. The highest was in the study of Ghosh et al. [25] with eight deaths in 315 children (2.5%). The most common cause of mortality was head injuries. Three studies described no deaths [20,32,75].

### 4.3. Changes Over Time

Davidson et al. [33] noted a decrease in equestrian-related injuries in the winter months, as noted in this study. We noted an annual percentage decrease of 3.64% in ED visits for children with fractures due to equine activities (Figure 7a) from 2020 through 2023. This may reflect a gradual decrease in the number of horses in the US. In 2003, there were 9.2 million horses in the US and in 2023 it had dropped to 6.6 million, or a 28.3% decrease [76]. This extrapolates to an annual percentage decrease of 1.4%. This may be due to increasing awareness and injury prevention/protective equipment.

### 4.4. Injury Prevention and Protective Equipment

As with all injuries, prevention is better than treatment. The long-term prognosis for children with equestrian injuries is not always good. In a questionnaire study of 100 patients < 17 years of age who had sustained an equestrian-related injury [77], 41 still had residual disabilities at a 4-year follow-up. Absence from school averaged 2 weeks and return to horse riding averaged 4 months, with 29% stopping horse riding completely. Results on the Child Health Questionnaire were poorer than those of a control group in many subscales (physical function, bodily pain, general behavior, mental health, and self-esteem). Risk factors for poor long-term outcomes were being an advanced rider, injuries other than extremity fractures, or sustaining subsequent injuries after the initial injury.

The history behind protective equipment began in 1964 when the United States Equestrian Federation began requiring protective headgear for all sanctioned jumping competitions [78]. Then, in 1990, helmets certified by the American Society for Testing and Materials became required for juniors, and in 2003 for adults. Back protectors for jumping were first recommended in 1994, and “body protecting vests” became mandatory for the cross-country phase of events in 1996. Indeed, the United States Pony club [79] mandates that every Pony Club member must wear a properly fitted, approved helmet with the chin harness secured at all times (1) when mounted, (2) when lounging, (3) at horse inspections (jog for soundness), and (4) at pre-riding inspections. Helmets are mandatory at all competition venues and most training facilities but are often not used at home. Protective vests are mandated for cross-country competitions but not for show jumping. There is no known effective protection against cervical spine injuries.

The majority of the literature regarding protective equipment focuses on the use of helmets. Most authors conclude that helmet use is protective [26,33,73,74,78,80,81]. Barone et al. [75], in a study of 136 patients ≤ 19 years of age, concluded that if effective safety gear had been utilized, many of the severe injuries could have been prevented. Moss et al. [80] noted that the majority of head-injured riders wearing approved helmets sustained only minor injuries. Bier et al. [81] noted that the odds ratio for a skull fracture was 8.14 times greater in the non-helmeted riders. Holland et al. [82] found that children wearing helmets when riding were less likely to suffer long-term neurological sequelae compared to those who did not.

The role of protective equipment outside of helmets is less clear. O’Farrell [38], in a series of nine pelvic and acetabular injuries caused by horse riding, stressed the importance of wearing appropriate protective clothing. However, Hessler et al. [26], in 2012, studied 31 patients who sustained torso injuries while horseback riding, with 61 control riders with injuries other than the torso. Safety vest use was not associated with a lower risk of torso injuries. They, however, tempered their findings by acknowledging that the development of a new generation of safety vests might be more effective but that further study is needed. They also noted that an effective vest adapted for children and adolescents should be developed and studied. In rodeo athletes [83], protective vests seem to have little impact on fatal thoracic injuries, while there were no reported cases of fatality due to head injury in those wearing helmets.

In this study, the vast majority of fractures involved the upper extremity, especially the wrist and forearm. Would protective equipment have reduced this number? Moss et al. in 2002 [80] noted that there was protective gear recommended for the upper limb and more specifically the wrist. This paper identifies the potential need for research and development of such protection. It must be remembered that wrist motion is very important to a horse rider, and any protective equipment for the wrist would likely reduce the amount of wrist motion and perhaps create/result in more falls. Maurel et al. [84] biomechanically studied a new design wrist guard which allowed for movement of the wrist and digits and found that the new guard demonstrated the same ability to reduce impact forces and absorb energy as commercially available fixed guards. To our knowledge, this has not been clinically tested.

Obstacles, perceived or real, to using protective equipment need to be mentioned. One is simply logistics. Ingemarson et al. [74] recommended that all riders with less experience should train in an indoor area supervised by a teacher; and even more importantly, those with 3 years’ experience or more should continue advanced riding together with a teacher. This greater supervision comes at a cost, both financial and in terms of teacher availability to the less experienced rider. Holland et al. [82] recommend that a standard approved helmet for horse riding should be made mandatory but noted it would not be practical to enforce helmet use in rural areas. However, compulsory use during competitions, in riding schools, and on public highways might have an effect on farms and in children who become occupational riders as adults.

Another obstacle, which could be overcome by education, is the lack of awareness on the part of parents regarding the dangers of horses to children. This was nicely shown by Shumway et al. [85], who studied Diné (Navajo) farmers’ and ranchers’ perceptions of injury risks to children who live or work in agricultural operations. This questionnaire study of 96 parents found that 63% perceived that youth who work with intact male livestock were at high risk for injury and that 26% perceived that youth who ride horses without equestrian helmets were at high risk for injury. Approximately, 96% agreed or strongly agreed that they would use an online training program to promote agricultural health and safety for their youth. When participants were asked if there were safety issues associated with having youth working on a farm or ranch, a very large portion felt that the biggest issue was a lack of education and instruction from elders. A recommendation for an injury prevention practice included developing a user-friendly online network, giving parents and community leaders access to resources to assist in educating youth in local agricultural traditions integrated with safety training. This likely extends to all parents, not just the Diné people.

Asa et al. [86] recommended, based on the results from their study, that the prevention of horseback-riding-related injuries should follow three guidelines:

(1) Adopt safe riding practices. Riders should always use protective equipment such as a helmet, safety vest, and gloves, in order to prevent injury and minimize injury severity.
(2) Be aware of the environment. Riders should be knowledgeable about the horse’s response(s) to various environmental conditions (e.g., weather, distractions, and riding inside vs. outside) in order to prepare and handle the horse accordingly.
(3) Be aware of the horse at all times. Riders should know where the horse’s head and legs are at all times and should ensure that the horse is in a good mental mindset before riding. Future research that identifies strategies to implement these recommendations is warranted. Prevention programs, especially targeted at females aged 15–19 years, that not only promote the use of protective equipment but also educate horseback riders on horse behavior, the proper handling of horses, and safe riding practices should be developed, implemented, and evaluated.

### 4.5. Strengths and Limitations

There are certainly limitations in this study using large, non-granular databases. As this is an ED-focused database, events occurring after admission to the hospital, primarily fatalities and long-term outcomes, are not known. It also does not capture minor injuries treated in physicians’ offices, urgent care centers, or not seen by a health care provider at all. While we found an open fracture rate of 1.1%, it could be higher, as those data were culled from the narrative comments in the database, and if not mentioned by the coder, then they would not have been identified. The same applies to those children with spinal fractures and a neurologic deficit; no deficits were identified, but that is again dependent upon the coder. However, in general, the accuracy of the NEISS database shows more than 90% accuracy [87,88]. More importantly, many researchers have emphasized that the NEISS data should be used for further injury research due to their accuracy [89,90,91]. The age cutoff for inclusion was solely dependent upon chronological age, as bone age was not available for these patients. However, in a large cohort of otherwise healthy children, the difference between chronological and bone age is likely minimal. Finally, the NEISS is US-nation-based, and researchers cannot hone in on state or city-wide levels due to privacy constraints; however, the hospital stratum classification does indirectly reflect urban vs. rural locations/hospitals, with the exception of the children’s hospital strata, which are nearly always urban-based but frequently receive children from rural settings. Also, these results may not apply to national studies from other nations. For instance, a study of horse-related injuries in 53 Israeli children [92] found that 79% were male, compared to the 27.5% male in this study.

The strengths of this study are that it spans 24 years, includes all patients seen in EDs from rural to large urban hospitals, and both those treated and released as well as admitted to the hospital. It is not limited to the more severe injuries such as those seen at designated trauma centers or studies called from national trauma data banks. It therefore gives the most accurate overall picture of the types of fractures seen in children due to equine activity. 

## 5. Conclusions

For the period 2000–2023, there were 101,677 children seen in EDs with an equestrian-related fracture. There was an overall admission rate of 18.5% from the ED to the hospital for these fractures. Fractures mostly involved the upper extremity (80.0%). The humerus, elbow, radius/ulna, and wrist accounted for 62.4% of all the fractures. The youngest age group (0- to 5-year-olds) had more boys and was more likely to be admitted to the hospital. This extensive description of fracture patterns due to equestrian injuries in children can be used to determine the effectiveness of future prevention strategies, such as protective equipment and educational programs. Further studies should be performed in the future to assess new prevention programs and protective equipment. Finally, this study gives pediatric trauma and orthopedic surgeons an overall picture of the types of fractures which occur in children due to equestrian activities.

## Figures and Tables

**Table 1:** General demographics.

Parameter	n	N	L95%	U95%	%
Hospital size					
Small	665	48,338	39,146	57,692	47.5
Medium	276	48,338	39,146	57,692	47.5
Large	255	14,949	7656	27,179	14.7
Very large	810	12,982	8195	19,970	12.8
Children	1156	6654	3244	13,188	6.5
Sex			0	0	0.0
Male	897	28,131	24,382	32,212	27.7
Female	2265	73,546	69,466	77,296	72.3
Race					
White	2209	74,348	71,015	76,617	92.0
Black	67	1852	1229	2789	2.3
Other	194	4628	2481	8430	5.7
ED disposition					
Released	2436	82,906	79,279	86,057	81.6
Admitted	724	18,708	15,557	22,335	18.4
Incident Locale					
Home	709	27,393	21,508	33,655	42.0
Farm/ranch	244	10,069	6139	15,846	15.4
Street	27	870	502	1507	1.3
Other pub property	124	4018	2929	5473	6.2
School	7	141	46	444	0.2
Sports/rec	752	27	28,606	28,801	0.0
Injury mechanism					
Fall	2489	76,755	72,595	80,485	76.0
Bucked/thrown/kicked off	461	17,416	13,860	21,649	17.2
Step/stomped on	77	2801	2020	3869	2.8
Kicked by horse	46	1431	808	2526	1.4
Tack malfunction	24	1185	727	1909	1.2
MVC	3	38	10	121	0.0
Bitten by horse	1	5	0	40	0.0
Struck	29	1016	606	1707	1.0
Misc	10	376	172	828	0.4
Mounting					
On horse	3070	97,863	96,540	98,824	96.8
Not on horse	48	1995	1202	3304	2.0
Mounting	7	450	212	960	0.4
Dismounting	16	729	414	1273	0.7
Spooked					
Yes	63	2268	1403	3630	2.2
No	3099	99,410	98,048	100,275	97.8
Events					
Yes	32	1096	590	2023	1.1
No	3130	100,581	99,655	101,088	98.9
Dragged by horse					
Yes	18	372	193	722	0.4
No	3144	101,306	100,956	101,485	99.6
Tack involvement					
Yes	71	2679	1891	3782	2.6
No	3091	98,998	97,896	99,787	97.4
Internal organ injury					
Yes	114	2846	1393	5724	2.8
No	3048	98,831	95,954	100,285	97.2

n = actual number, N = estimated number, L95% and U95% = the lower and upper 95% confidence intervals of the estimate.

**Table 2:** Detailed fracture location.

Fracture Location	N	%
Radius/ulna	26,423	27.5
Wrist	19,196	14.6
Humerus	11,902	11.2
Elbow	8025	9.1
Clavicle	6881	6.0
Tibia/fibula	3607	3.8
Skull	2589	3.7
Shoulder	3596	3.1
Finger	3201	2.8
Face	2000	2.7
Ankle	2775	2.7
Femur	1819	2.3
Hand	1881	1.5
Pelvis	1311	1.5
Foot	1388	1.3
Rib	1208	1.3
Lumbar spine	1305	1.2
Thoracic spine	1032	1.1
Sacrococcygeal spine	517	0.5
Hip	192	0.5
Cervical spine	205	0.4
Toe	585	0.4
Scapula	288	0.3
Knee	203	0.3

**Table 3:** Differences between appendicular and axial fracture locations.

Variable	Appendicular					Axial					
	n	N	L95%	U95%	%	n	N	L95%	U95%	%	*p* Value
All	2808	92,767	90,285	94,728	91.7	339	8437	6477	10,920	8.3	
Age (years)	10.2 [10.0, 10.3]					11.2 [10.6, 11.7]					0.0004
Stratum											
Small	622	45,200	36,968	53,508	48.7	40	2922	1920	4237	34.6	0.021
Medium	255	17,341	11,614	25,029	18.7	20	1336	769	2200	15.8	
Large	222	12,924	7106	22,264	13.9	31	1814	580	4544	21.5	
Very large	726	11,637	7291	18,034	12.5	82	1314	771	2130	15.6	
Children	983	5666	2829	11,002	6.1	166	952	375	2175	11.3	
Sex											
Male	800	25,615	22,227	29,314	27.6	89	2150	1544	2895	25.5	0.53
Female	2008	67,152	63,453	70,540	72.4	250	6287	5542	6893	74.5	
Race											
White	1964	68,342	65,567	70,265	92.2	235	5686	5097	6016	89.6	0.64
Black	61	1675	1097	2557	2.3	6	177	63	479	2.8	
Other	173	4087	2275	7188	5.5	20	485	183	1189	7.6	
ED disposition											
Released	2282	78,668	80,355	85,143	80.5	144	3987	3054	4942	47.3	0.0003
Admitted	525	14,040	12,565	17,353	14.4	194	4446	3490	5378	52.7	
Incident Locale											
Home	630	25,174	19,698	30,992	42.1	78	2141	1503	2834	42.3	0.63
Farm/ranch	223	9329	5608	14,842	15.6	19	613	293	1196	12.1	
Street	20	613	334	1111	1.0	7	258	102	620	5.1	
Other pub property	109	3679	2622	5125	6.2	14	324	148	681	6.4	
School	6	124	36	448	0.2	1	17	2	124	0.3	
Sports/rec	670	20,808	16,025	26,166	34.8	76	1708	1068	2493	33.8	
Injury mechanism											
Fall	2261	71,533	67,997	74,684	77.7	217	4906	4195	5578	58.1	0.021
Bucked/thrown/kicked off	379	14,847	11,965	18,266	16.1	80	2495	1784	3344	29.6	
Step/stomped on	67	2601	1851	3638	2.8	9	195	69	532	2.3	
Kicked by horse	22	819	433	1557	0.9	23	534	238	1147	6.3	
Tack malfunction	23	1102	663	1824	1.2	1	83	11	584	1.0	
MVC	2	32	9	120	0.0	1	7	1	46	0.1	
Bitten by horse	1	5	0	37	0.0	0	0	0	0	0.0	
Struck	21	798	442	1437	0.9	8	218	95	490	2.6	
Misc	10	376	166	829	0.4	0	0	0	0	0.0	
Mounting											
On horse	2732	89,564	88,473	90,344	97.1	324	7914	7310	8181	94.6	0.014
Not on horse	34	1535	931	2508	1.7	13	381	140	992	4.6	
Mounting	6	379	157	894	0.4	1	72	10	494	0.9	
Dismounting	16	729	415	1291	0.8	0	0	0	0	0.0	
Spooked											
Yes	54	1811	1048	3117	2.0	8	451	219	901	5.3	0.082
No	2754	90,957	89,650	91,719	98.0	331	7986	7536	8218	94.7	
Open injury											
Yes	24	944	529	1679	1.0	10	198	102	381	2.3	0.11
No	2784	91,824	91,088	92,238	99.0	329	8239	8056	8335	97.7	
Events											
Yes	28	998	566	1763	1.1	3	94	19	455	1.1	0.96
No	2780	91,770	91,004	92,201	98.9	336	8344	7982	8418	98.9	
Dragged by horse											
Yes	12	198	93	417	0.2	6	174	51	577	2.1	0.13
No	2796	92,570	92,350	92,674	99.8	333	8263	7860	8386	97.9	
Tack involvement											
Yes	64	2404	1670	3451	2.6	4	188	51	658	2.2	0.81
No	2744	90,373	89,316	91,097	97.4	335	8249	7779	8386	97.8	

n = actual number, N = estimated number, L95% and U95% = the lower and upper 95% confidence intervals of the estimate.

**Table 4:** Differences by sex in fractures due to equine activities.

Variable	Male					Female					
	n	N	L95%	U95%	%	n	N	L95%	U95%	%	*p* Value
	897	28,131	24,382	32,212	27.7	2265	73,546	69,466	77,296	72.3	-
Age (years)	9.9 [9.6, 10.2]					10.4 [10.2, 10.6]					0.004
Hospital size											
Small	217	15,663	12,094	18,848	55.7	448	32,675	26,131	39,494	44.4	0.10
Medium	55	3660	2068	6189	13.0	221	15,095	10,149	21,623	20.5	
Large	59	3497	1162	8968	12.4	196	11,452	6524	19,048	15.6	
Very large	199	3192	1845	5331	11.3	611	9786	5781	15,908	13.3	
Children	367	2116	785	5263	7.5	789	4538	2434	8237	6.2	
Race											
White	588	19,060	17,446	20,160	86.7	1621	55,288	53,410	56,547	94.0	0.003
Black	37	1037	624	1697	4.7	30	816	483	1365	1.4	
Other	89	1884	914	3701	8.6	105	2744	1536	4831	4.7	
ED disposition											
Released	663	22,350	21,003	23,496	79.6	1773	60,556	57,891	62,848	82.3	0.15
Admitted	233	5723	4576	7069	20.4	491	12,985	10,693	15,650	17.7	
Incident Locale											
Home	261	9709	7676	11,696	52.6	448	17,684	13,538	22,252	37.8	0.003
Farm/ranch	81	3232	979	2787	17.5	163	6837	4112	10,909	14.6	
Street	7	203	14	89	1.1	20	668	374	1179	1.4	
Other pub property	31	1180	9	19	6.4	93	2838	1993	4004	6.1	
School	1	17	0	8	0.1	6	124	33	444	0.3	
Sports/rec	135	4116	3	5	22.3	617	18,627	14,450	23,150	39.8	
Injury mechanism											
Fall	681	20,020	18,538	21,355	71.4	1808	56,734	53,672	59,431	77.1	0.035
Bucked/thrown/kicked off	139	5491	4305	6909	19.6	322	11,925	9299	15,117	16.2	
Step/stomped on	23	867	513	1458	3.1	54	1933	1292	2876	2.6	
Kicked by horse	18	419	202	860	1.5	28	1013	533	1912	1.4	
Tack malfunction	6	227	78	647	0.8	18	958	569	1599	1.3	
MVC	1	16	2	23	0.0	0	0	0	0	0.0	
Bitten by horse	0	0	1	5	0.0	0	0	0	0	0.0	
Struck	16	656	13	360	0.2	1	345	847	5394	0.5	
Miscellaneous	7	334	3	790	1.2	3	42	7	190	0.1	
Mounting											
On horse	865	27,018	26,323	27,421	96.7	2205	70,856	69,792	71,583	96.9	0.83
Not on horse	18	676	321	1403	2.4	30	1318	768	2252	1.8	
Mounting	2	83	14	525	0.3	5	367	154	863	0.5	
Dismounting	5	167	53	525	0.6	11	562	278	1126	0.8	
Spooked											
Yes	10	402	177	897	1.4	53	1866	1052	3273	2.5	0.28
No	887	27,729	27,234	27,954	98.6	2212	71,681	70,273	72,494	97.5	
Open											
Yes	9	291	115	734	1.0	25	851	427	1699	1.2	0.86
No	888	27,840	27,397	28,016	99.0	2240	72,695	71,847	73,119	98.8	
Events											
Yes	9	344	124	951	1.2	23	753	412	1361	1.0	0.73
No	888	27,788	27,180	28,007	98.8	2242	72,794	72,185	73,134	99.0	
Dragged by horse											
Yes	7	151	62	369	0.5	11	221	81	581	0.3	
No	890	27,980	27,762	28,069	99.5	2254	73,325	72,965	73,465	99.7	0.43
Tack involvement											
Yes	17	598	324	1094	2.1	54	2081	1397	3082	2.8	0.40
No	880	27,533	27,037	27,807	97.9	2211	71,465	70,464	72,149	97.2	

n = actual number, N = estimated number, L95% and U95% = the lower and upper 95% confidence intervals of the estimate.

**Table 5:** Differences in mechanism by the 5 major fracture locations.

	Upper Extremity					Lower Extremity					Spine					Skull/Face					Chest (Rib/Sternum)					
Variable	n	N	L95%	U95%	%	n	N	L95%	U95%	%	n	N	L95%	U95%	%	n	N	L95%	U95%	%	n	N	L95%	U95%	%	*p* Value
Injury mechanism																										
Fall	1998	64,786	61,810	67,389	80.5	263	6747	5758	7682	57.2	58	1483	987	1990	48.5	136	2864	2427	3252	64.5	23	559	343	742	59.5	<10-4
Bucked/thrown/kicked off	328	13,129	10,668	16,030	16.3	51	1718	1138	2523	14.6	37	1329	806	1905	43.4	37	852	538	1288	19.2	6	313	141	551	33.3	
Step/stomped on	19	554	314	982	0.7	48	2047	1430	2855	17.4	1	5	1	38	0.2	4	137	35	504	3.1	4	53	18	144	5.6	
Kicked by horse	9	407	185	894	0.5	13	412	198	838	3.5	2	75	11	453	2.5	20	444	206	900	10.0	1	15	2	98	1.6	
Tack malfunction	19	880	515	1506	1.1	4	222	73	654	1.9	1	83	12	510	2.7	0	0	0	0	0.0	0	0	0	0	0.0	
Mounting																										
On horse	2358	78,940	78,023	79,510	98.2	374	10,624	10,001	11,045	90.1	101	2990	2587	3051	97.7	189	3985	3550	4199	91.2	34	940	0	0	100.0	0.0001
Not on horse	17	857	474	1544	1.1	17	678	370	1214	5.8	1	70	9	473	2.3	12	311	125	724	7.1	0	0	0	0	0.0	
Mounting	5	309	121	885	0.4	1	70	9	503	0.6	0	0	0	0	0.0	1	72	10	484	1.6	0	0	0	0	0.0	
Dismounting	8	313	113	860	0.4	8	417	191	886	3.5	0	0	0	0	0.0	0	0	0	0	0.0	0	0	0	0	0.0	

n = actual number, N = estimated number, L95% and U95% = the lower and upper 95% confidence intervals of the estimate.

**Table 6:** Differences between the three age groups.

Variable	0 to 5 Years					6 To 10 Years					11 to 15 Years					
	n	N	L95%	U95%	%	n	N	L95%	U95%	%	n	N	L95%	U95%	%	*p* Value
All	394	12,153	10,032	14,659	11.7	1155	36,185	33,600	38,861	34.8	1670	55,623	52,979	58,249	53.5	-
Hospital size																
Small	102	7400	5656	8942	60.9	240	17,454	13,942	21,013	48.2	338	24,605	19,457	29,986	44.2	0.0003
Medium	14	899	534	1483	7.4	104	7138	4556	10,689	19.7	164	11,154	7442	16,097	20.1	
Large	28	1726	614	4131	14.2	78	4434	2305	8062	12.3	156	9236	4756	16,565	16.6	
Very large	67	1072	611	1825	8.8	286	4578	2869	7089	12.7	468	7509	4683	11,647	13.5	
Children	338	1057	4251	6552	8.7	447	2580	1321	4874	7.1	544	3120	1557	6063	5.6	
Sex																
Male	170	4878	4221	5566	40.1	298	9259	7657	11,069	25.6	443	14,465	12,126	17,076	26.0	<10^−4^
Female	224	7275	6587	7932	59.9	857	26,926	25,116	28,528	74.4	1227	41,158	38,547	43,497	74.0	
Race																
White	277	8873	10,685	11,565	73.0	823	27,446	32,031	34,524	75.8	1151	39,896	48,125	52,853	71.7	0.74
Black	9	233	108	783	1.9	20	486	279	1259	1.3	39	1138	934	2253	2.0	
Other	29	508	372	1089	4.2	74	1683	1147	3615	4.7	93	2469	1463	6564	4.4	
Disposition from the ED																
Released	280	8982	8158	9688	73.9	890	30,069	28,402	31,521	83.1	1309	45,589	43,341	47,508	82.0	0.029
Admitted	114	3170	2465	3995	26.1	264	6057	4664	7783	16.7	360	10,030	8115	12,282	18.0	
Incident Locale																
Home	119	3887	4450	8113	32.0	247	9678	11,702	18,433	26.7	356	14,309	16,909	28,167	25.7	0.092
Farm/ranch	36	1738	1541	4686	14.3	75	3081	2913	7519	8.5	134	5268	4728	13,572	9.5	
Street	4	119	51	698	1.0	9	295	192	1071	0.8	14	456	367	1368	0.8	
Other pub property	12	343	242	1236	2.8	56	1654	1712	3770	4.6	59	2233	2403	4995	4.0	
School	1	7	1	74	0.1	3	36	18	185	0.1	4	103	33	740	0.2	
Sports/rec	41	1402	1474	3370	11.5	298	8674	10,233	16,916	24.0	426	13,313	15,602	26,477	23.9	
Injury mechanism																
Fall	333	9962	9220	10,548	82.0	950	29,302	27,866	30,739	81.0	1241	38,837	36,188	42,023	69.8	0.031
Bucked/thrown/kicked off	30	1385	930	2022	11.4	144	4701	3528	6235	13.0	290	11,465	9033	14,646	20.6	
Step/stomped on	10	330	119	881	2.7	20	757	423	1361	2.1	47	1714	1185	2520	3.1	
Kicked by horse	11	134	36	478	1.1	16	653	365	1165	1.8	19	645	278	1507	1.2	
Tack malfunction	4	153	43	538	1.3	8	310	134	767	0.9	18	944	601	1513	1.7	
MVC	0	0	0	15	0.0	0	0	0	145	0.0	3	38	11	122	0.1	
Bitten by horse	1	17	2	128	0.1	0	0	0	0	0.0	1	5	0	39	0.0	
Struck	1	5	1	35	0.0	4	178	54	575	0.5	28	1066	662	1741	1.9	
Misc	4	169	49	572	1.4	5	132	36	470	0.4	8	313	133	751	0.6	
Fracture Location																
Appendicular	342	10,805	10,548	11,530	88.9	1043	33,473	33,004	34,734	92.5	1423	48,489	48,564	51,351	87.2	0.011
Axial	43	981	623	1605	8.1	95	2124	1451	3181	5.9	201	5332	4272	7059	9.6	
Upper extremity	312	10,016	9612	10,870	82.4	953	30,889	30,196	32,389	85.4	1142	40,068	39,503	43,158	72.0	<10^−4^
Lower extremity	30	789	514	1269	6.5	90	2584	1921	3564	7.1	281	8421	7531	10,040	15.1	
Spine	1	60	10	403	0.5	18	471	271	843	1.3	83	2529	1858	3654	4.5	
Skull/face	40	830	514	1398	6.8	64	1370	865	2222	3.8	99	2238	1691	3143	4.0	
Rib/sternum	2	91	18	468	0.7	13	283	130	633	0.8	19	566	317	1085	1.0	

n = actual number, N = estimated number, L95% and U95% = the lower and upper 95% confidence intervals of the estimate.

## Data Availability

These data are publicly available online at https://www.cpsc.gov/cgibin/NEISSQuery/home.aspx. Further details regarding the NEISS data and the acquisition and guidelines for its use are available at www.cpsc.gov/library/neiss.html.

## Supplementary Materials

The following supporting information can be downloaded at https://www.mdpi.com/article/10.3390/children11121443/s1, Supplemental Table S1: Comparisons between the five major fracture locations.
